# A Hybrid Preprocessing Multi-Objective Surrogate Model for Thermal MEMS Actuators

**DOI:** 10.3390/mi17060755

**Published:** 2026-06-22

**Authors:** Armin Aghajani, Ali Nazari, Phiona Buhr, Byoungyoul Park, Yunli Wang, Cyrus Shafai

**Affiliations:** 1 Department of Electrical and Computer Engineering, University of Manitoba, Winnipeg, MB R3T 5V6, Canada; aghajana@myumanitoba.ca (A.A.); nazaria@myumanitoba.ca (A.N.); buhrdphb@myumanitoba.ca (P.B.); 2 Quantum and Nanotechnologies Research Centre, National Research Council Canada, Edmonton, AB T6G 2M9, Canada; byoungyoul.park@nrc-cnrc.gc.ca; 3 Digital Technologies Research Centre, National Research Council Canada, Ottawa, ON K1A 0R6, Canada; yunli.wang@nrc-cnrc.gc.ca

**Keywords:** MEMS thermal actuators, multi-objective optimization, surrogate modeling, Gaussian Process Regression (GPR), ensemble learning, finite element method (FEM)

## Abstract

In this study, an advanced surrogate model is proposed to simultaneously predict five key output variables, including deformation, stress, temperature, current density, and resonance frequency. This study used two models: Gaussian Process Regression (GPR) and an ensemble model based on Random Forest and XGBoost. By generating 10,000 design samples using the Latin Hypercube sampling method and performing simulations in COMSOL Multiphysics, as well as applying eight preprocessing methods, GPR achieved a mean absolute percentage error (MAPE) between 0.81% and 2.58%, whereas the ensemble model’s MAPE ranged from 3.05% to 9.20%. The ensemble model offers substantially faster training, whereas GPR achieves higher prediction accuracy across all output variables. Additionally, a 5-fold cross-validation scheme was implemented to ensure reliable model evaluation. This surrogate model, achieving multi-objective prediction with strong scalability due to efficient preprocessing and sampling strategies, is an effective step in reducing computational costs and accelerating the design process of MEMS actuators.

## 1. Introduction

Micro-electromechanical systems (MEMS) have found applications across fields such as biomedical engineering and aerospace technologies due to their compact size, low power consumption, and high sensitivity [1]. With the increasing integration of MEMS devices into modern technologies, they play an increasingly important role in systems that bridge the physical and digital domains, such as the Internet of Things [2]. More recently, MEMS-based electro-optical transducers have also been explored for emerging quantum technologies, including quantum computing and quantum sensing [3]. The design of MEMS components requires accurate prediction of device behavior, which is commonly achieved through multiphysics finite element method (FEM) simulations. However, such simulations are computationally expensive, particularly when exploring large design spaces or performing sensitivity analyses. This challenge is amplified by the strong coupling between mechanical, electrical, thermal, and fluidic domains at micro-scales, where analytical modeling is often ineffective [4]. Consequently, there is a growing need for fast and accurate predictive tools to support efficient early-stage design exploration [5].

To overcome this computational burden, surrogate models trained on high-fidelity FEM data have been widely investigated as efficient alternatives. These models approximate system responses with significantly reduced computational cost once trained. Despite substantial progress in this area [6,7,8], most existing approaches remain limited in scope, often focusing on a small number of output variables or relying on problem-specific formulations. Furthermore, surrogate models are frequently embedded within optimization frameworks, which limits their standalone applicability for general prediction tasks.

From a methodological perspective, prior work on surrogate modelling for MEMS can be categorized into three main strands. The first strand includes machine-learning-based surrogate models tailored to specific data representations. For instance, convolutional neural network (CNN)-based models have been used to predict electrostatic MEMS motor responses such as torque and radial force with high accuracy [9]. These approaches demonstrate strong predictive capability but are typically dependent on structured spatial inputs, which limits their applicability to general tabular design problems.

The second strand consists of Gaussian Process Regression (GPR)-based surrogate models applied to MEMS components such as micro-actuators and resonators [4]. While these methods provide reliable predictions under uncertainty, they are generally restricted to a small number of input variables and limited output dimensions, which reduces their scalability to multi-output design problems.

The third strand comprises surrogate-assisted optimization frameworks that integrate surrogate models with evolutionary algorithms such as NSGA-II or adaptive sampling strategies [10,11]. These approaches improve efficiency within constrained design spaces; however, the surrogate models are typically evaluated within the optimization loop and are often trained using limited datasets with uniform preprocessing strategies, which may reduce robustness and standalone predictive performance.

In addition, several studies have highlighted the effectiveness of surrogate models for repeated simulations and design exploration outside optimization loops [12,13,14]. In such cases, the primary computational benefit arises from replacing expensive FEM evaluations rather than improving optimization efficiency. Recent advances in machine learning–based surrogate models have further demonstrated their ability to capture nonlinear relationships in high-dimensional MEMS design spaces [15], thereby improving accuracy while maintaining computational efficiency. Across these strands, several key limitations remain particularly relevant to thermal MEMS actuators, including limited simultaneous prediction of multiple performance outputs, restricted training-data coverage, insufficient handling of heterogeneous output distributions, and limited evaluation of standalone surrogate performance. A structured summary of these categories and their limitations is provided in Table 1.

From a practical perspective, full-scale FEM simulations remain computationally prohibitive for iterative MEMS design and uncertainty analysis. Therefore, surrogate models are particularly valuable in early-stage design, where rapid evaluation of design alternatives is required [16]. Although training these models requires an initial computational cost, this cost is amortized over subsequent predictions, which are significantly faster than FEM simulations.

To address these limitations, this work proposes a scalable multi-output surrogate modelling pipeline for thermal MEMS actuators. The framework is developed using 10,000 COMSOL-generated samples, eight objective-specific preprocessing strategies, and two regression models: Gaussian Process Regression and an ensemble model combining Random Forest and XGBoost. Unlike prior studies that typically consider one or two output variables or simplified modeling assumptions [4,6,7,8,9,10,11], the proposed framework simultaneously predicts five output variables: deformation, stress, temperature, current density, and resonance frequency. This unified formulation enables efficient exploration of a wide and highly nonlinear design space while ensuring both predictive accuracy and computational scalability. Table 1 summarizes the main categories of prior work and clarifies the methodological positioning of the present study.

## 2. Problem Definition

**Figure 1:**
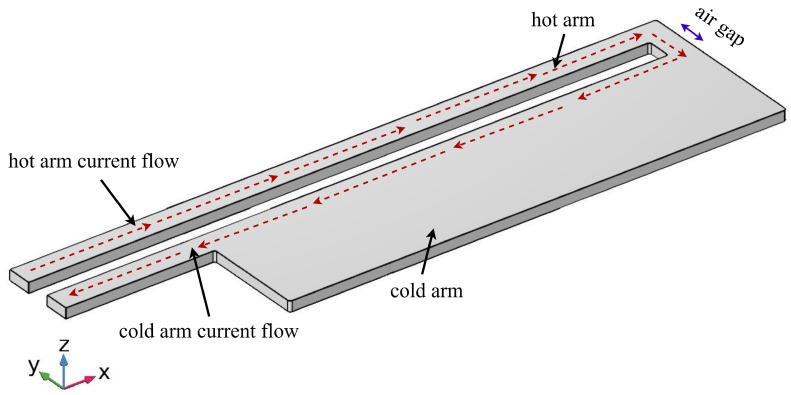
Schematic of the thermal actuator showing dimensions and direction of motion.

This study focuses on the surrogate modeling of a specific class of microelectromechanical thermal actuators known as Single Hot Arm Thermal Actuators. In our design, the actuator is made of polycrystalline silicon, a typical but not exclusive choice in MEMS, and operates in microscale environments by exploiting thermal expansion differences between asymmetrically heated arms to induce mechanical deformation. The actuator design consists of four main arms: the hot arm, the cold arm, the flexure, and the junction arm. All arms are structurally connected at the junction arm, which transmits the displacement resulting from differential thermal expansion between the hot and cold arms. A schematic of the thermal actuator, illustrating its dimensions and direction of motion, is shown in Figure 1.

**Figure 2:**
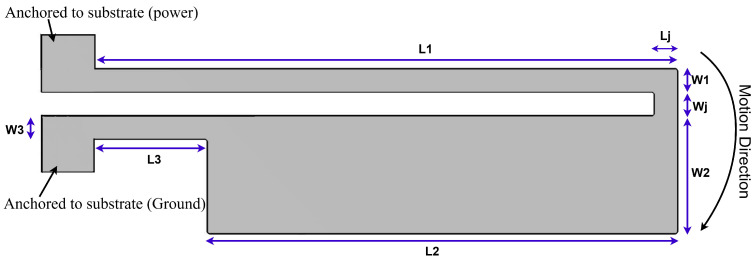
Detailed schematic of the thermal actuator, highlighting key geometric features used as design variables.

When a current is passed through the actuator between the two fixed pads, higher Joule heating in the thinner active beam causes its temperature to increase, while the temperature of the cold arm remains relatively unchanged. This differential thermal expansion between the two arms induces mechanical displacement, which is transmitted through the junction arm. Both the hot and cold arms are anchored to fixed pads, with the cold arm connected through an additional passive beam, a flexure. A detailed schematic of a typical thermal actuator, highlighting its key geometric features used as design variables, is provided in Figure 2.

The geometric configuration of these arms, particularly the thermal asymmetry between the hot and cold arms, governs the resulting mechanical displacement and directly influences key performance parameters such as deformation, stress, resonance frequency, current density, and thermal response.

To design this actuator, we focus on ten key design variables: the lengths and widths of the arms ($L_{1}$, $L_{2}$, $L_{3}$, $L_{j}$, $W_{1}$, $W_{2}$, $W_{3}$, $W_{j}$), and the applied voltage (AV). The actuator thickness is held constant across the device and is defined as the overall thickness ($t_{\mathrm{all}}$). All design variables are listed in Table 2 and were chosen because they directly affect the actuator’s performance, including deformation, stress handling, thermal response, current density, and dynamic behavior through resonance frequency. The anchors’ dimensions and behavior are neglected in this study. Our model aims to predict five outcomes, shown in Table 3, with predefined goals for each. To ensure feasible and efficient designs, the following constraints are applied to the design variables:

(1) $$W_{2}>W_{1},\;W_{2}>W_{3},\;L_{1}=L_{2}+L_{3},\;L_{2}>L_{j}$$

These constraints establish the relationships among the design parameters and ensure that the resulting actuator configurations are physically feasible and perform effectively.

## 3. Methodology

**Figure 3:**
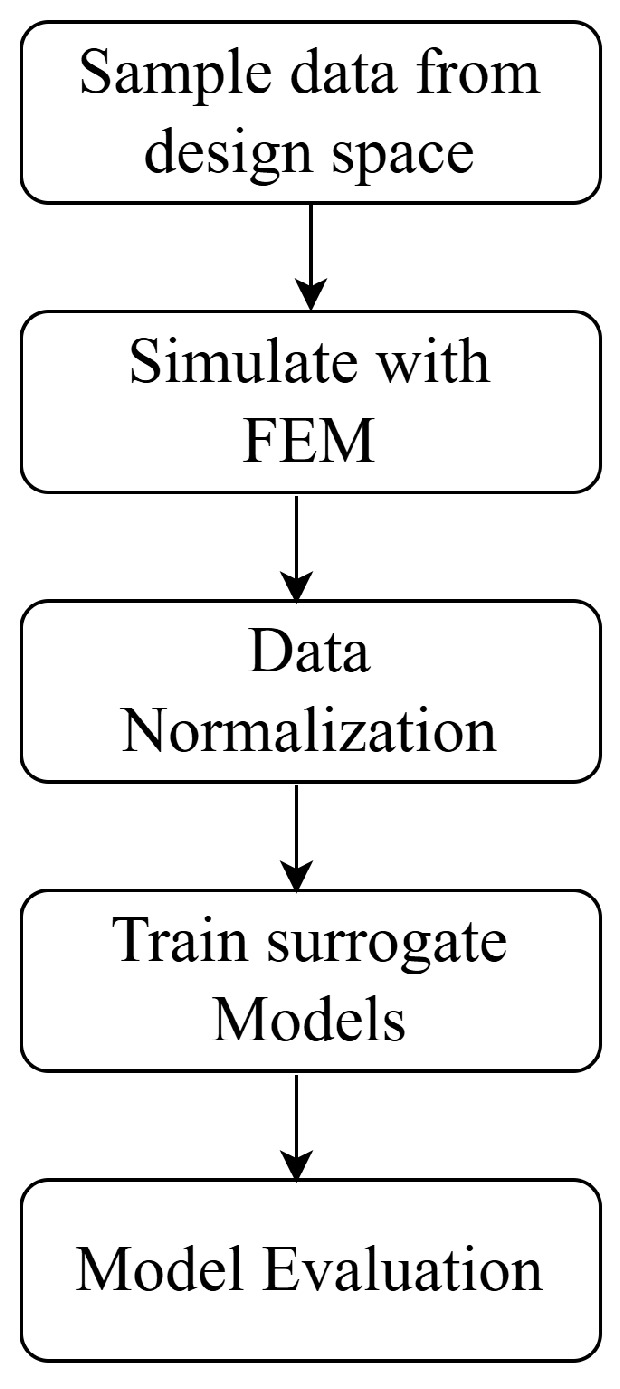
Flowchart of Surrogate Model Development for Thermal Actuator.

This section explains the steps of developing a surrogate model for simulating a MEMS thermal actuator. The flowchart of the surrogate model development process is shown in Figure 3. This process includes data generation from the design space utilizing FEM simulation, data preprocessing, and model training and evaluation. Each step is described below.

### 3.1. Sampling Data from Design Space

To ensure robust predictive performance, the surrogate model requires a training dataset that adequately and uniformly covers the design space. For this reason, Latin Hypercube Sampling (LHS) is employed in this study, given its strong space-filling capability and efficiency in high-dimensional design problems. As a stratified sampling technique, LHS ensures that values of each input variable are sampled across its full distribution range while maintaining statistical independence between variables, making it particularly suitable for engineering optimization and surrogate modeling applications [17].

To further enhance the uniformity of sample distribution, the maximin criterion is applied to maximize the minimum distance between sample points, thereby reducing clustering and improving coverage of the design space. All geometric dimensional variables, such as $L_{1}$, $L_{2}$, $L_{3}$, $L_{j}$, $W_{1}$, $W_{2}$, $W_{3}$, and $W_{j}$, were sampled independently within predefined intervals based on the defined range for each variable. Meanwhile, the thickness $t_{\mathrm{all}}$ and the applied voltage AV were kept constant at 5μm and 5V, respectively. In total, this work utilizes the LHS with the maximin criterion to generate 10,000 design samples.

Table 4 compares LHS with other commonly used sampling techniques to justify its selection [17,18,19].

### 3.2. Finite Element Method (FEM)

COMSOL Multiphysics^®^ software, version 6.1, was used for the numerical simulations (COMSOL AB, Stockholm, Sweden, 2022). Finite element method (FEM) simulations were performed on a Mac Studio equipped with an M1 Ultra processor and 64 GB of RAM, using a multiphysics interface that couples electrical, thermal, and structural domains. COMSOL Multiphysics^®^ was chosen for this study due to its native multiphysics coupling capabilities, which enable seamless integration of electrical, thermal, and structural domains without external solvers.

In the model, both the hot and cold arms are clamped at their anchor pads, imposing zero-displacement boundary conditions. The cold arm is connected to its anchor via a flexure, and the junction between the arms converts differential thermal expansion into output displacement. A constant voltage is applied across the anchor pads to drive current through the beams, with Joule heating in the polysilicon acting as the sole heat source. Heat transfer is modeled exclusively by conduction to the silicon substrate; further details are provided in Section 3.3.

The governing equations include the heat conduction equation, Ohm’s law for electrical conduction, and the structural mechanics equations under thermal loading. To accurately resolve steep temperature gradients near the anchors and along current-carrying segments, a physics-controlled, extra-fine mesh is employed. The polycrystalline silicon structure is modeled as homogeneous and isotropic, using standard material properties (density, Young’s modulus, thermal conductivity, etc.), which are assumed to be temperature-independent over the operating range. Anchor structures themselves are not explicitly modeled; instead, they are simplified as fixed boundary conditions.

### 3.3. Material Properties and Modeling Assumptions

The actuator is modeled in polycrystalline silicon, chosen for its high stiffness and thermal conductivity. Material properties are drawn from standard MEMS fabrication values, and the thermal modeling only considers heat conduction to the substrate. Radiative and convective heat transfer are not included in the model, as explained below. In heat transfer, energy moves through conduction, convection, and radiation. In this work, the only heat source is Joule heating in the poly-Si beams, and heat mainly leaves the device through solid-state conduction through the anchors into the silicon substrate [20,21]. Convective and radiative losses were left out of the model. This was not because they are always negligible; in fact, they may matter at larger device sizes, but because including them would have added more variables to an already large design space. The model was built around geometry and applied voltage as the main inputs, so these effects were intentionally set aside.

To quantify the validity of neglecting convection, two dimensionless parameters are evaluated [22]. The Biot number $\left(\mathrm{Bi}=hL_{c}/k\right)$, which compares the resistance to surface convective heat loss to internal conduction within the solid, is estimated using the device half-thickness as characteristic length $L_{c}=t/2=2.5\;\mu m$ (where t=5μm is the device thickness), polysilicon thermal conductivity $k=34\;W\;m^{-1}\;K^{-1}$, and a free-convection coefficient h=5–$25\;W\;m^{-2}\;K^{-1}$:

(2) $$\mathrm{Bi}=\frac{hL_{c}}{k}\approx 3.7\times 10^{-7}\;\mathrm{to}\;1.8\times 10^{-6}\ll 0.1$$

This confirms that across the solid thickness, surface convective losses are negligible relative to internal conduction. The Grashof number $\left(\mathrm{Gr}=g\beta \Delta TL^{3}/\nu^{2}\right)$, which quantifies the ratio of buoyancy to viscous forces in the surrounding fluid, is estimated using the maximum surface-to-ambient temperature difference ΔT=1280K (corresponding to the simulated temperature range of 20–1300 °C), $\beta \approx 1/T_{f}$ (thermal expansion coefficient of air as an ideal gas) at a representative film temperature $T_{f}\approx 950\;K$, $\nu \approx 1.5\times 10^{-4}\;m^{2}\;s^{-1}$ (kinematic viscosity of air at $T_{f}$), and $L=L_{1}$ as the dominant device characteristic length (500–10,000μm per Table 1):

(3) $$\mathrm{Gr}=\frac{g\beta \Delta TL^{3}}{\nu^{2}}\approx 0.19\;\left(\mathrm{at}\;L=500\;\mu m\right)\;\mathrm{to}\;1520\;\left(\mathrm{at}\;L=10,000\;\mu m\right)$$

Although Gr remains small at the lower end of the design space, it increases substantially at larger device scales, indicating that buoyancy-driven convection cannot be rigorously neglected throughout the full geometric range of the surrogate model. For radiation, assuming emissivity ε=0.7 as a representative value for polysilicon surfaces [22], the linearized radiative coefficient $h_{\mathrm{rad}}=4\varepsilon \sigma T^{3}$ (where $\sigma =5.67\times 10^{-8}\;W\;m^{-2}\;K^{-4}$ is the Stefan–Boltzmann constant) gives $h_{\mathrm{rad}}\approx 34$–$650\;W\;m^{-2}\;K^{-1}$ over the simulated temperature range T≈600–1600K. The corresponding radiative Biot number is:

(4) $${\mathrm{Bi}}_{\mathrm{rad}}=\frac{h_{\mathrm{rad}}L_{c}}{k}\approx 2.5\times 10^{-6}\;\mathrm{to}\;4.8\times 10^{-5}\ll 0.1$$

The radiative Biot number remains negligible across the full design space, confirming that radiation is a genuinely secondary heat loss mechanism relative to conduction. Convective effects, however, particularly at larger device scales, represent a more significant simplification, and their influence on predicted outputs should be considered accordingly.

Meshing is performed using a physics-controlled, extra-fine resolution to ensure accurate numerical representation of thermal gradients and structural deformation [20]. This resolves steep temperature gradients near the anchors and along current-carrying segments. The polycrystalline silicon structure in this actuator is modeled as homogeneous and isotropic. Its mechanical and thermal properties include a Young’s modulus of $160\times 10^{9}\;\mathrm{Pa}$, Poisson’s ratio of 0.22, density of $2320\;\mathrm{kg}\;m^{-3}$, heat capacity at constant pressure of $678\;J\;{\left(\mathrm{kg}\;\cdot \;K\right)}^{-1}$, and thermal conductivity of $34\;W\;{\left(m\;\cdot \;K\right)}^{-1}$. Additionally, the electrical resistivity was assumed to be $1\times 10^{-4}\;\Omega \;\cdot \;m$, corresponding to an electrical conductivity of $1\times 10^{4}\;S/m$. This electrical conductivity was used to calculate the current density and Joule heating under an applied voltage of 5V. All these material properties were held constant over the simulated temperature range. Although the Young’s modulus, thermal conductivity, electrical resistivity, and thermal expansion coefficient of polycrystalline silicon are known to vary with temperature [23,24,25], incorporating temperature-dependent properties would significantly increase the complexity of the already large design space. Future work can incorporate temperature-dependent E(T), k(T), $\rho_{e}\left(T\right)$, and α(T) to improve the physical fidelity of the model.

### 3.4. Surrogate Modeling

This section outlines the surrogate modeling framework implemented in this study. We utilize two regression-based surrogate models: Gaussian Process Regression (GPR) and an ensemble learning model that combines Random Forest Regression and XGBoost. These models are chosen for their ability to effectively capture nonlinear relationships between design features and the five performance outputs. The framework includes dataset generation, preprocessing strategies, model formulation, training procedures, and evaluation metrics used to assess predictive accuracy and generalization performance.

#### 3.4.1. Data Preprocessing

To ensure robust statistical behavior and reliable regression accuracy, eight preprocessing techniques were employed on the input and output variables. These methods were specifically chosen to address distributional issues frequently encountered in simulated MEMS datasets, including skewness, heavy tails, and outliers, which can adversely affect model fitting, distort error propagation, and diminish generalization performance.

- MinMax: Scales data to [0, 1] based on minimum and maximum values, preserving data distribution.
  (5) $$x^{\prime }=\frac{x-x_{\min}}{x_{\max}-x_{\min}}$$
  where *x* denotes the original FEM simulation output for a given objective, $x^{\prime }$ represents the corresponding normalized value, and $x_{\min}$ and $x_{\max}$ are the minimum and maximum values of *x* computed over the training dataset.
- Z-Score: Standardizes data to zero mean and unit variance, assuming a Gaussian-like distribution.
  (6) $$x^{\prime }=\frac{x-\mu_{x}}{\sigma_{x}}$$
  where $\mu_{x}$ and $\sigma_{x}$ are the mean and standard deviation of the data.
- Robust: Scales data using the median and interquartile range (IQR), providing robustness against outliers.
  (7) $$x^{\prime }=\frac{x-\mathrm{median}(x)}{\mathrm{IQR}(x)}$$
  where *x* denotes the original FEM simulation output, $x^{\prime }$ represents the scaled value, and IQR(x) is the interquartile range. This is defined as the difference between the third quartile ($Q_{3}$, 75th percentile) and the first quartile ($Q_{1}$, 25th percentile), i.e., $\mathrm{IQR}=Q_{3}-Q_{1}$, which quantifies the statistical dispersion of the data.
- Quantile Normal: Transforms data to follow a standard normal distribution using quantile mapping, reducing skewness.
  (8) $$x^{\prime }=\Phi^{-1}\left(\frac{\mathrm{rank}(x)-0.5}{n}\right)$$
  where $\Phi^{-1}$ is the inverse cumulative distribution function of the standard normal distribution, rank(x) is the rank of *x*, and *n* is the number of samples.
- Log + MinMax: Applies a natural logarithm transformation to stabilize the variance, followed by MinMax to scale the data to map it to [0, 1]. This is suitable for data that has a skewed distribution.
  (9) $$x_{\log}=\ln\left(x+\epsilon \right),\;x^{\prime }=\frac{x_{\log}-x_{\log,\min}}{x_{\log,\max}-x_{\log,\min}}$$
  where ϵ is a small constant to avoid ln(0), and $x_{\log,\min}$ and $x_{\log,\max}$ are the minimum and maximum log-transformed values.
- Log + Robust: Applies a log transformation followed by Robust scaling, which uses the median and interquartile range (IQR) to handle outliers.
  (10) $$x_{\log}=\ln\left(x+\epsilon \right),\;x^{\prime }=\frac{x_{\log}-\mathrm{median}\left(x_{\log}\right)}{\mathrm{IQR}(x_{\log})}$$
  where $\mathrm{IQR}=Q_{3}-Q_{1}$, with $Q_{1}$ and $Q_{3}$ denoting the first and third quartiles, respectively.
- Log + Z-Score: Applies a log transformation followed by standardization to zero mean and unit variance. (Suitable for data with skewed distribution and a need for standardization).
  (11) $$x_{\log}=\ln\left(x+\epsilon \right),\;x^{\prime }=\frac{x_{\log}-\mu_{x_{\log}}}{\sigma_{x_{\log}}}$$
  where $\mu_{x_{\log}}$ and $\sigma_{x_{\log}}$ are the mean and standard deviation of the log-transformed data.
- Yeo–Johnson: A power transformation that handles both positive and negative values, stabilizing variance, and makes data more Gaussian-like.
  (12) $$x^{\prime }=\left\{\begin{array}{cc} \frac{{\left(x+1\right)}^{\lambda }-1}{\lambda }, & \lambda \neq 0,\;x\geq 0,\\ \ln(x+1), & \lambda =0,\;x\geq 0,\\ -\frac{{\left(-x+1\right)}^{\;2-\lambda }}{2-\lambda }, & \lambda \neq 2,\;x<0,\\ -\ln(-x+1), & \lambda =2,\;x<0, \end{array}\right.$$
  where λ is a parameter optimized to maximize the normality of the transformed data, ensuring that both positive and negative values become approximately Gaussian.

Scaling parameters are stored for consistent application to test data.

#### 3.4.2. Gaussian Process Regression

In this section, the surrogate model was implemented using Gaussian Process Regression (GPR) because this method can model complex nonlinear relationships between design variables and performance outputs and provides uncertainty estimation, which is therefore well-suited for MEMS actuators with expensive simulations. In this study, GPR was implemented using the Scikit-learn library in Python [26] with the following configuration described below:

The Matérn kernel with a smoothing parameter of v = 1.5 was chosen because it is flexible enough to model relationships with moderate variations and balances sensitivity to data fluctuations with noise resistance. A low-noise WhiteKernel was also added to the model to account for the small amount of noise in the simulated data. To predict five output variables (deformation, stress, temperature, current density, and resonance frequency), a MultiOutputRegressor was employed, training an independent GPR model for each output variable. This approach, commonly used in previous multi-output surrogate modeling studies, enables output-specific preprocessing and noise modeling, thereby improving adaptability and prediction accuracy for output variables with heterogeneous distributions and scales. Then, a custom optimizer based on the L-BFGS-B algorithm was used to tune the core hyperparameters. Since L-BFGS-B is a gradient-based method, it can effectively explore the hyperparameter space by imposing constraints on the parameters and preventing overfitting, which can improve the accuracy of model predictions. To estimate the model’s expected performance and minimize the risk of overfitting, 5-fold cross-validation was employed. In this procedure, the dataset was divided into five subsets, and in each iteration, the model was trained on four subsets and evaluated on the remaining one. This approach enhances the model’s robustness to data variability and leads to more reliable results. The mathematical formulation of Gaussian Process Regression (GPR) is as follows. Let f(x) be a latent function defined as a Gaussian process: $f\left(x\right)∼\mathrm{GP}\left(0,k(x,x^{\prime })\right)$, and the observed output is given by: $y=f\left(x\right)+\epsilon ,\;\epsilon ∼N\left(0,\sigma_{n}^{2}\right)$. The covariance between function values at *x* and $x^{\prime }$ is defined by the Matérn kernel:

(13) $$k\left(x,x^{\prime }\right)=\sigma_{f}^{2}\frac{2^{1-v}}{\Gamma (v)}{\left(\frac{\sqrt{2v}∥x-x^{\prime }∥}{\ell }\right)}^{v}K_{v}\left(\frac{\sqrt{2v}∥x-x^{\prime }∥}{\ell }\right)$$

where $\sigma_{f}^{2}$ is the signal variance, *ℓ* is the length-scale hyperparameter, v=1.5 controls smoothness, Γ(·) is the gamma function, and $K_{v}\left(\;\cdot \;\right)$ is the modified Bessel function of the second kind.

Given a training set $\left\{X_{t},y_{t}\right\}$ where $X_{t}\in R^{n\times d}$ and $y_{t}\in R^{n}$, the predictive distribution for new inputs $X_{*}$ is Gaussian with mean and covariance:

(14) $$\mu_{*}=K_{*t}{\left(K_{t}+\sigma_{n}^{2}I\right)}^{-1}y_{t}$$

(15) $$\Sigma_{*}=K_{**}-K_{*t}{\left(K_{t}+\sigma_{n}^{2}I\right)}^{-1}K_{t*}$$

where $K_{*t}$ is the covariance between test and training inputs, $K_{t}$ is the covariance matrix of training inputs, $K_{t*}$ is its transpose, and $I\in R^{n\times n}$ is the identity matrix. The hyperparameters $\theta =\{\ell ,\sigma_{f}^{2},\sigma_{n}^{2}\}$ are optimized by maximizing the log marginal likelihood:

(16) $$\log p\left(y|X,\theta \right)=-\frac{1}{2}y^{T}K_{y}^{-1}y-\frac{1}{2}\log\left|K_{y}\right|-\frac{n}{2}\log2\pi$$

where: $K_{y}=K_{t}+\sigma_{n}^{2}I$

#### 3.4.3. Ensemble Model

In this study, the ensemble method is used to combine two machine learning models, random forest regression (RF) [27] and XGBoost [28], to improve the accuracy of predictions and minimize generalization error. Ensemble models [29] are beneficial when the data are nonlinear, noisy, or have other complex information structure features, because by combining the strengths of several different models, a balance between bias and variance can be achieved, and the overall performance of the system can be improved. The selection of RF and XGBoost was motivated by several factors. Both are tree-based methods capable of efficiently capturing nonlinear relationships and interactions in the data, which are common in complex MEMS datasets. Compared to GPR, which provides high accuracy but incurs substantial computational cost for large datasets due to cubic scaling with the number of data points, RF and XGBoost are considerably faster while maintaining comparable predictive performance.

In this study, RF was configured with 200 decision trees and a maximum depth of 10, whereas XGBoost was constructed with 200 weak learners and a maximum depth of 6. These hyperparameters were selected to achieve an appropriate trade-off between prediction accuracy and computational efficiency. Only two models were included in the ensemble to maintain simplicity and interpretability, as preliminary tests indicated that adding additional models did not significantly improve performance.

The high speed and efficiency of these models result from the way their underlying algorithms process data and construct predictions. RF constructs multiple decision trees in parallel, allowing for independent and rapid computation, and aggregates their predictions to reduce variance. XGBoost sequentially improves weak learners through gradient boosting while utilizing optimized tree-building algorithms and regularization techniques to prevent overfitting and enhance computational speed. In contrast to GPR, which requires inversion of large covariance matrices, these tree-based methods perform simple and scalable computations, explaining their superior efficiency.

The predictions from the two models were combined using a simple averaging approach to obtain the final output. This process was evaluated using the same 5-fold cross-validation procedure previously described for GPR. During preliminary experiments, weighted averaging, boosting, and stacked generalization (meta-learning) were also considered alongside simple averaging. However, these more sophisticated ensemble strategies did not yield noticeable improvements in prediction accuracy compared to simple averaging, while simultaneously increasing model complexity and reducing interpretability. Consequently, to maintain computational efficiency, simplicity, and model transparency, simple averaging was chosen as the final ensemble strategy. The final output is derived by averaging the predictions of the two models as follows:

(17) $$y_{\mathrm{ensemble}}=\frac{y_{\mathrm{RF}}+y_{\mathrm{XGBoost}}}{2}$$

where $y_{\mathrm{RF}}$ and $y_{\mathrm{XGBoost}}$ represent the predictions from the Random Forest and XGBoost models, respectively.

#### 3.4.4. Metric

In this study, Mean Absolute Percentage Error (MAPE) was selected as the primary evaluation metric due to the differing physical units and numerical scales of the five performance targets. MAPE offers a scale-independent percentage error, enabling fair comparisons across these heterogeneous outputs and improving the interpretability of regression accuracy. This metric is defined as follows:

- MAPE: Mean Absolute Percentage Error (MAPE) measures the average absolute percentage difference between the actual and predicted values. It is defined as follows:
  (18) $$\mathrm{MAPE}\left(\%\right)=\frac{100}{n}\sum_{i=1}^{n}\left|\frac{y_{i}-{\hat{y}}_{i}}{y_{i}}\right|$$
  where $y_{i}$ denotes the actual value, ${\hat{y}}_{i}$ denotes the predicted value, and *n* represents the total number of samples. MAPE expresses the prediction error as a percentage, making it suitable for comparing model performance across datasets with different scales. A lower MAPE value indicates higher prediction accuracy, with a value of 0% corresponding to perfect predictions.

## 4. Results and Discussion

In this section, two surrogate modeling approaches are investigated and compared: GPR and an Ensemble model consisting of Random Forest Regression and XGBoost models. Their predictive performance is evaluated based on five key output variables of MEMS-based thermal actuators: deformation, stress, temperature, current density, and resonance frequency. The data was split into training and test sets (80–20). A 5-fold cross-validation was applied to the training set, meaning that in each fold, 64% of the overall data was used for training and 16% for validation, while the 20% test set remained completely unseen until final evaluation. The Mean Absolute Percentage Error (MAPE) was used as the evaluation metric during cross-validation. Subsequently, the model was evaluated on the test set using this same metric, consistent with the methodology section. In addition to standard quantitative metrics, parity plots and absolute relative error distribution plots provide a more detailed assessment of surrogate model performance and prediction accuracy.

### 4.1. Trends of Test MAPE for Model Performance Evaluation

**Figure 4:**
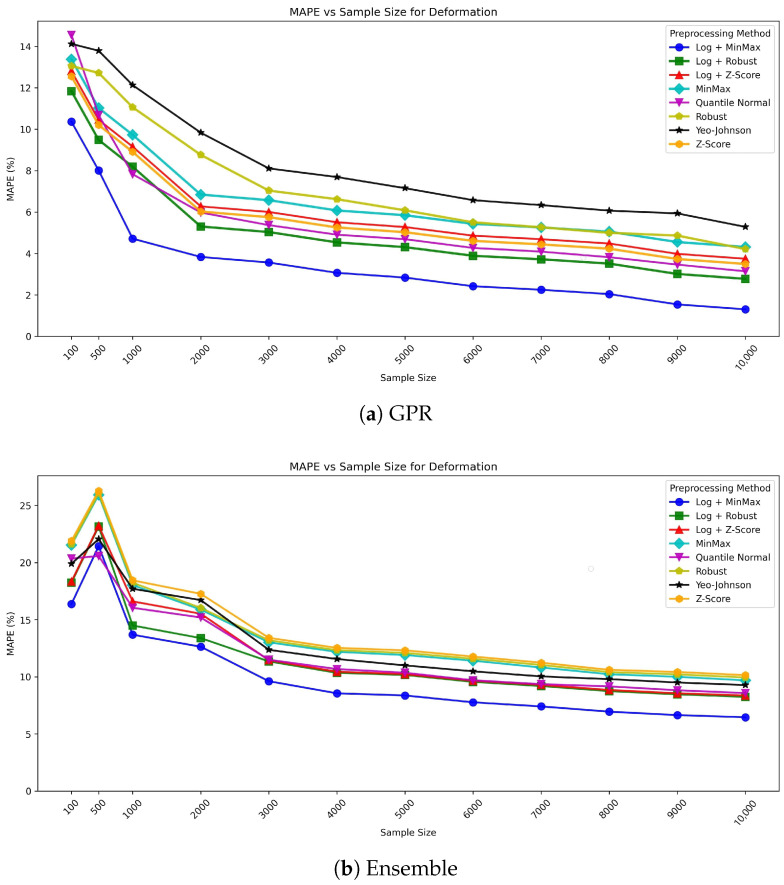
MAPE vs. Number of samples at 8 preprocessing methods in Deformation for GPR and Ensemble models: (**a**) GPR model, (**b**) Ensemble model.

**Figure 5:**
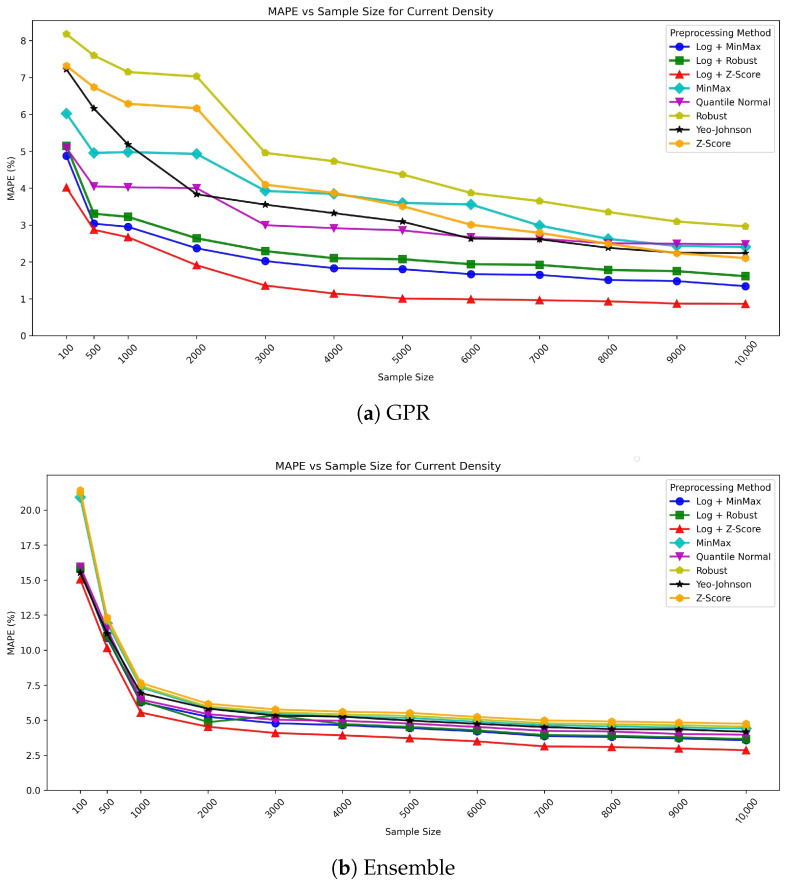
MAPE vs. Number of samples at 8 preprocessing methods in Current Density for GPR and Ensemble models. (**a**) GPR model, (**b**) Ensemble model.

**Figure 6:**
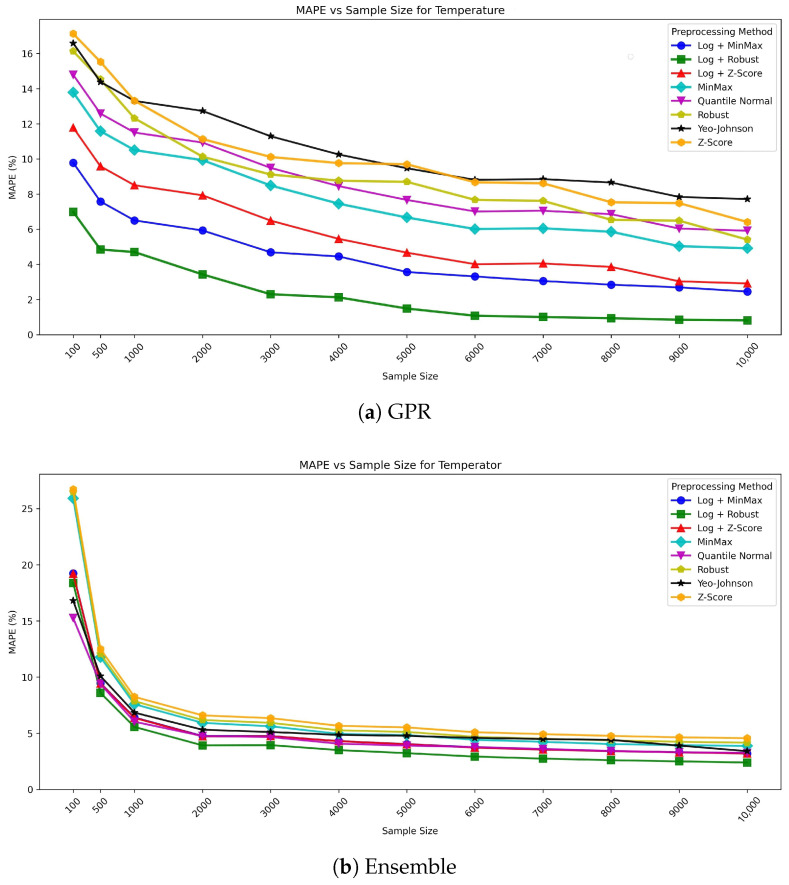
MAPE vs. Number of samples at 8 preprocessing methods in Temperature for GPR and Ensemble models. (**a**) GPR model, (**b**) Ensemble model.

**Figure 7:**
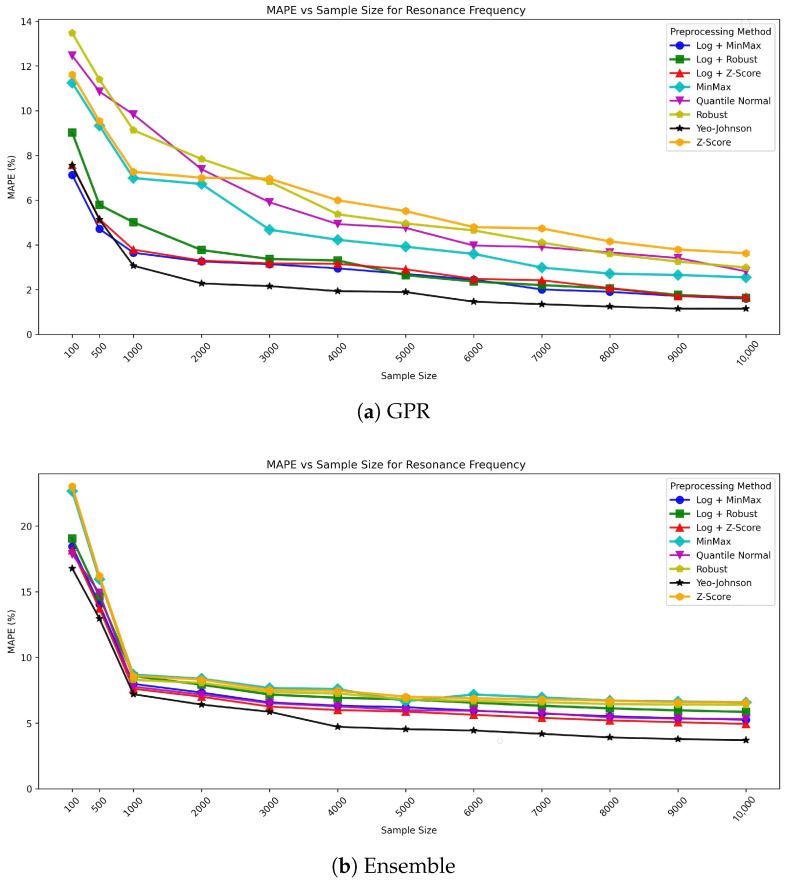
MAPE vs. Number of samples at 8 preprocessing methods in Resonance Frequency for GPR and Ensemble models. (**a**) GPR model, (**b**) Ensemble model.

**Figure 8:**
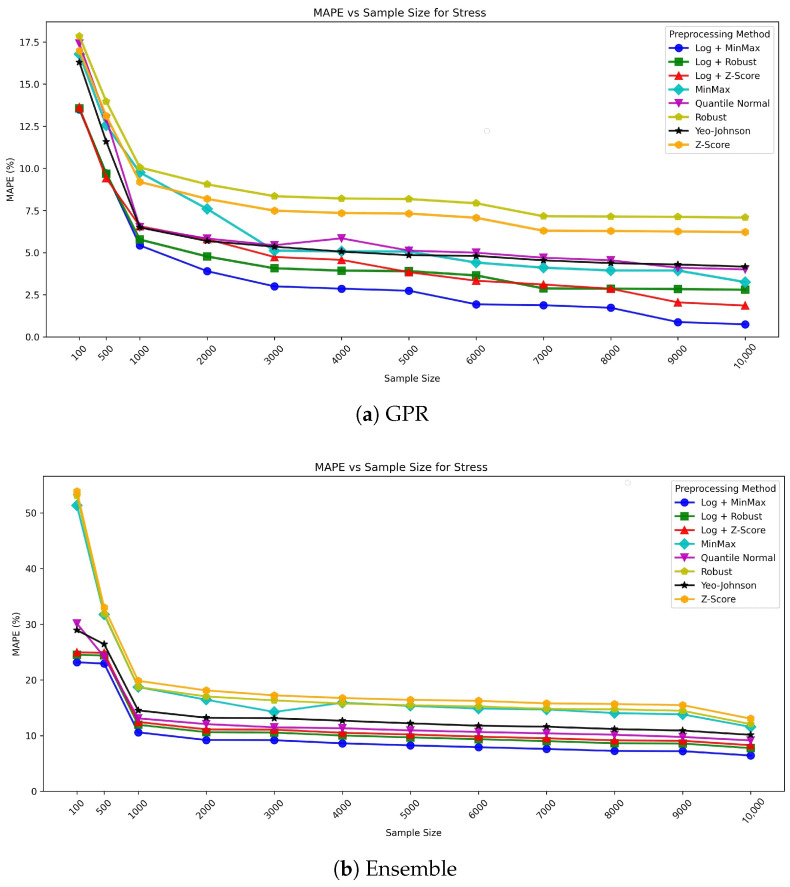
MAPE vs. Number of samples at 8 preprocessing methods in Stress for GPR and Ensemble models. (**a**) GPR model, (**b**) Ensemble model.

In this section, the changes in Test MAPE metrics are analyzed as the number of training samples increases. For each of the five output variables, namely deformation, stress, temperature, current density, and resonance frequency, the Test MAPE trends are presented. These trends were obtained using eight preprocessing methods across sample sizes ranging from 100 to 10,000. Figure 4, Figure 5, Figure 6, Figure 7 and Figure 8 illustrate the results for both Gaussian Process Regression (GPR) and Ensemble models, where dashed lines represent Test MAPE.

- Deformation: Figure 4 illustrates performance differences between the GPR and Ensemble models based on sample size and preprocessing methods. For the GPR model, the MAPE is approximately 11–14% at 100 samples and decreases as sample size increases. After 5000 samples, performance continues to improve slightly, though it is not fully stabilized. The Log + MinMax and Log + Robust methods achieve better performance, with MAPEs of 1.5% and 2.5% at 10,000 samples, respectively. This indicates that combining logarithmic transformation with robust scaling is more suitable for deformation prediction.
  The ensemble model begins with an initial MAPE of about 16–25% at 100 samples and decreases as the sample size increases. After 5000 samples, the model continues to improve gradually with minor fluctuations, reaching a MAPE of 7–13% at 10,000 samples. Specifically, Log + MinMax achieves the best result at 6.85% MAPE for 10,000 samples. Both models show improved performance with increasing sample sizes, with noticeable improvements continuing beyond 5000 samples. Within both models, the Log + MinMax and Log + Robust preprocessing methods consistently achieve the best performance.

- Current Density: Figure 5 clearly shows that the effectiveness of the GPR and Ensemble models in predicting current density depends largely on the sample size and the nature of the preprocessing. For the GPR model, the MAPE value decreases to 7–8% with 100 samples and to 4% after 5000 samples. When using this model, the Log + Z-Score method yields the best performance, achieving a MAPE of 1% at larger sample sizes. The Ensemble model, although starting with a high MAPE (approximately 20%), improves to approximately 2.5–3% with 10,000 samples. In this model, Log + Z-Score also yields the best result (3% MAPE), although its accuracy remains slightly lower than that of the GPR at the largest sample size.

In general, both models improve with larger amounts of data; however, the rate of improvement changes slightly beyond 5000 samples, indicating that performance does not fully stabilize. The GPR model with Log + Z-Score demonstrates the highest accuracy at large sample sizes, while the Ensemble model offers a relative advantage in terms of stability and robustness to the type of preprocessing. Therefore, Log + Z-Score is confirmed as the most effective preprocessing method for both models.

- Temperature: As shown in Figure 6, analysis of the Temperature variable reveals performance differences between the GPR and Ensemble models, influenced by sample size and preprocessing methods. For the GPR model, the Mean Absolute Percentage Error (MAPE) is approximately 15–16% at 100 samples, decreasing significantly as the sample size increases. This trend persists, and beyond 5000 samples, performance shows minor fluctuations within the 2–9% range. At 10,000 samples, the Log + MinMax and Log + Robust methods yield the best performance, with MAPEs of approximately 2.45% and 0.81%, respectively. Specifically, the Log + Robust method, combining logarithmic transformation with robust scaling, proves highly effective for temperature prediction.

The Ensemble model begins with an initial MAPE of approximately 15–25% at 100 samples, which decreases with increasing sample size. Beyond 5000 samples, it continues to show slight improvements, with MAPEs generally reaching 5–7%. Its optimal performance, a 3% MAPE, is achieved at 10,000 samples using the Log + Robust configuration. Both the Ensemble and GPR models improve with increasing sample size, showing marginal but non-negligible changes beyond 5000 samples.

- Resonance Frequency: Analysis of the Resonance Frequency plots for the GPR and Ensemble models, Figure 7, reveals performance differences based on sample size and preprocessing methods. For the GPR model, the Mean Absolute Percentage Error (MAPE) is approximately 8–14% at 100 samples and decreases significantly with increasing sample size. This decrease continues; beyond 5000 samples, performance fluctuates within the 2–6% range. Among the preprocessing methods, Yeo–Johnson demonstrates the best performance for GPR, achieving an MAPE of 1% at 10,000 samples.

The Ensemble model starts with an initial MAPE of approximately 17–25% at 100 samples and decreases with increasing sample size. Beyond 5000 samples, the model continues to show slight improvements, reaching an MAPE of 5–7%. Notably, Yeo–Johnson achieves 3.45% at 10,000 samples within this model. Both models exhibit improved performance with increasing sample size, with marginal but non-negligible changes continuing beyond 5000 samples.

- Stress: Figure 8 displays the graphs of the Stress variable for the two GPR models and the ensemble model. The GPR model’s Mean Absolute Percentage Error (MAPE) starts at approximately 14–17.5% with 100 samples and decreases significantly as the sample size increases. This improvement continues; after 5000 samples, its performance is within the 4–8% range for some preprocessing methods, though not yet fully stabilized. However, some methods still show a slight decrease in MAPE up to 10,000 samples. Among the Log + MinMax, Log + Z-Score, and Log + Robust methods, Log + MinMax demonstrates the best performance at 10,000 samples, achieving a MAPE approximately 1% lower than the others. This indicates that the combination of logarithmic transformation with robust scaling is more effective for stress prediction.

The ensemble model also begins with an initial MAPE of about 25–55% at 100 samples and decreases with increasing sample size. After 5000 samples, the model continues to exhibit slight improvements, reaching a MAPE of 10–15%, with Log + MinMax achieving the lowest MAPE of 8% at 10,000 samples. Both models demonstrate improved performance with increasing sample size.

### 4.2. Comparison of Ensemble Combination Strategies

The ensemble model used in this study combines the predictions from Random Forest and XGBoost regressors using a simple averaging strategy. While this straightforward formulation is adopted as the final model, we also investigated alternative ensemble strategies, including weighted averaging, boosting-based combination, and stacked generalization (meta-learning), to justify the selection of the final approach. To ensure a fair comparison, all strategies were evaluated using the same dataset of 10,000 samples and identical train-test partitions. Table 5 summarizes the resulting prediction errors (MAPE) for all target variables along with the corresponding training times.

As shown in Table 5, the simple averaging strategy achieved the best overall performance, producing the lowest prediction errors for all five output variables with an average MAPE of 5.46%. In comparison, boosting, weighted averaging, and stacking resulted in higher average MAPEs of 7.12%, 7.79%, and 8.59%, respectively. From a computational perspective, simple averaging was also the most efficient approach, requiring only 5.61 min of training time. In contrast, weighted averaging, boosting, and stacking required 7.12, 7.35, and 8.58 min, respectively. Thus, the more sophisticated ensemble strategies did not improve predictive accuracy and, at the same time, increased computational cost and model complexity. Based on these results, simple averaging was selected as the final ensemble strategy for the remainder of this study.

### 4.3. Effect of Training Set Size on Prediction Accuracy

To justify the use of 10,000 FEM samples, we compared model performance between 5000 and 10,000 training samples (Table 6). This extension was primarily conducted to verify the convergence and stability of the surrogate models. It allowed us to confirm that the stabilization of prediction error observed around 5000 samples was not temporary, and to assess whether further FEM simulations would continue to improve predictive performance or if the models had already approached a stable error region. The sample-size analysis showed that for the GPR model, the mean MAPE decreased from 2.7% (5000 samples) to 1.50% (10,000 samples), an improvement of 1.21 percentage points. Similarly, the Ensemble model’s mean MAPE decreased from 6.66% to 5.46%, representing a 1.20 percentage point improvement. However, the additional improvement beyond 5000 samples was relatively limited considering the increased computational cost of additional FEM simulations and training. Therefore, while approximately 5000 samples might offer a practical balance between computational cost and predictive accuracy, 10,000 samples were ultimately used to maximize robustness and fully verify convergence behavior.

### 4.4. Trends of Changes in the Training and Validation MAPE Criterion

**Figure 9:**
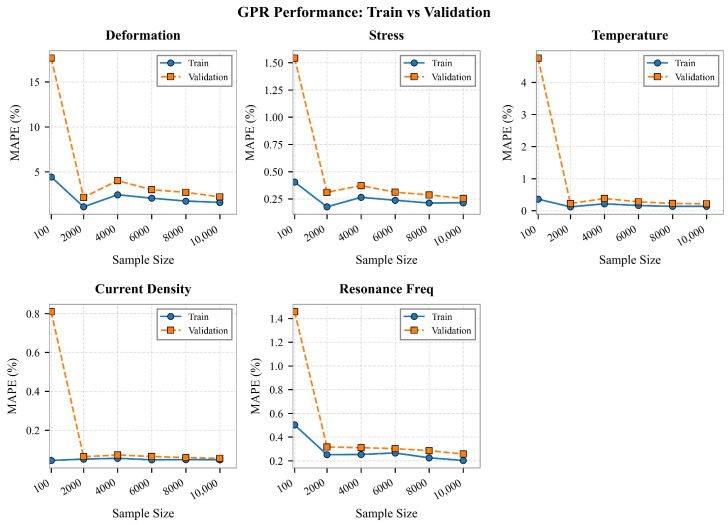
GPR model performance comparison across different output variables: Training vs. Validation.

**Figure 10:**
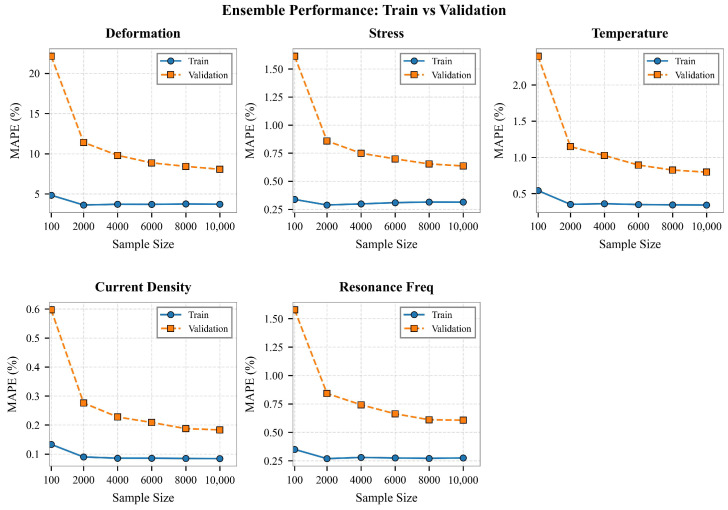
Ensemble model performance comparison across different output variables: Training vs. Validation.

Figure 9 and Figure 10 present the Training and Validation MAPE trends of the GPR and Ensemble models, respectively, for the five output variables (Deformation, Stress, Temperature, Current Density, and Resonance Frequency) across sample sizes ranging from 100 to 10,000. To reduce visual complexity and enhance figure clarity, only the best-performing scaling method for each objective was selected for inclusion.

For both models, the MAPE values consistently decrease as the number of training samples increases, indicating that larger sample sizes lead to improved model performance. The gap between Training and Validation MAPE remains relatively small for both models, suggesting that neither model suffers from significant overfitting.

The Ensemble model (Figure 10) exhibits a slightly larger, though still acceptable, gap between Training and Validation MAPE. This indicates good generalization without overfitting and stable performance across all sample sizes. In contrast, the GPR model (Figure 9) demonstrates superior generalization capability, reflected by a closer alignment between its Training and Validation curves. Furthermore, GPR consistently achieves lower MAPE values across all Ensemble model performance comparison across output variables and sample sizes, establishing it as the more accurate predictor for this application.

### 4.5. Computational Time Analysis and Model Selection

The GPR model demonstrates higher accuracy than the Ensemble model in predicting all variables, evidenced by lower MAPE values (e.g., 0.81% for temperature and 1.03% for Current Density). However, GPR’s computational cost is significantly higher. Table 7 provides a detailed breakdown of computational times, encompassing FEM simulation, model training, and total costs. For readability, FEM simulation time and total computational times are reported in hours, while both Ensemble and GPR training times are reported in minutes. For 10,000 samples, FEM simulation alone takes 27.75 h. GPR training adds another 1861.34 min, leading to a total computational time of 58.77 h. In contrast, the Ensemble model requires only 5.61 min for training, resulting in a total cost of 27.84 h when combined with FEM simulation. Notably, for 5000 samples, the total computational cost (FEM + Ensemble training) is 13.74 h, while the total cost (FEM + GPR training) is 18.19 h. Doubling the dataset from 5000 to 10,000 samples increases the total cost by a factor of 2.03 for the Ensemble model (13.74 to 27.84 h) and 3.23 for the GPR model (18.19 to 58.77 h). This confirms that GPR, despite its higher accuracy, suffers from a much steeper cost increase as data grows. Hence, while GPR is more accurate, the Ensemble model offers superior computational efficiency, making it a more practical choice for large-scale applications where time constraints matter.

In addition to training time, we compared the inference (test) time of both models on a fixed test set of 2000 unseen samples. Table 8 summarizes the average inference time and prediction accuracy for each of the five output variables. The ensemble model recorded a total inference time of 0.206 s, approximately 79 times faster than the GPR model (16.315 s). Although the GPR model yields lower MAPE values across all output variables, the ensemble model offers significantly faster prediction speed with acceptable accuracy, making it more suitable for applications requiring rapid surrogate evaluations.

### 4.6. Prediction Performance Evaluation Using Parity and Absolute Error Plots and Residual Error Distribution Plots

In this section, parity plots, absolute relative error distribution plots, and residual error distribution plots are used to provide a detailed assessment of surrogate model performance and prediction accuracy. The final surrogate models evaluated in this section were trained using the 10,000-sample dataset. For the visual performance assessment, these trained models were applied to a separate 7000-sample evaluation dataset generated independently using the same Latin Hypercube Sampling strategy. This 7000-sample dataset was not included in the training of the final 10,000-sample models and is therefore treated as unseen data for the analyses presented in this section.

The parity plots compare the surrogate model predictions with the corresponding COMSOL simulation results, providing a visual assessment of overall prediction agreement. The absolute relative error distribution plots evaluate the magnitude and distribution of sample-by-sample prediction errors across the design space, while the residual error distribution plots analyze the distribution of prediction residuals (Actual vs. Predicted) to assess prediction bias, symmetry, and error variability. Therefore, all parity plots, absolute relative error distribution plots, and residual error distribution plots presented in this section are generated using the independent 7000-sample evaluation dataset.

#### 4.6.1. Parity Plots for Key Output Variables

To qualitatively assess the predictive performance of the surrogate models, parity plots were generated for the five output variables using the evaluation dataset described in Section 4.6. In these plots, the horizontal axis represents the COMSOL simulation results, while the vertical axis represents the corresponding surrogate model predictions. Points closer to the diagonal reference line indicate stronger agreement between the surrogate model and COMSOL results, whereas deviations from this line indicate underprediction or overprediction.

**Figure 11:**
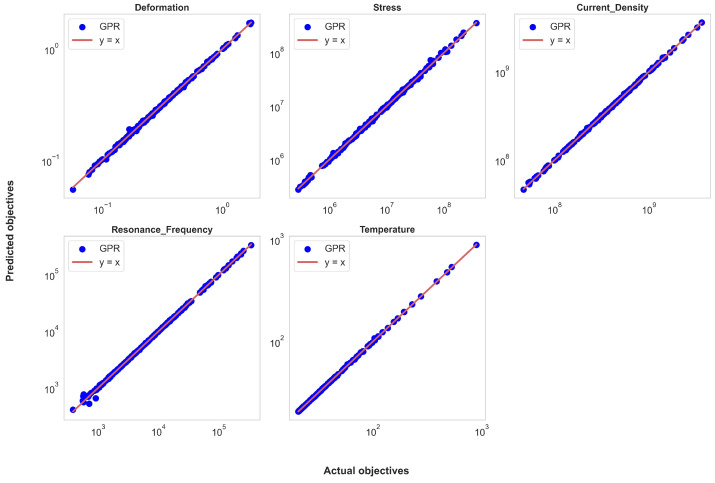
Parity plots: GPR-based surrogate model vs. COMSOL results (five output variables).

As shown in Figure 11, the GPR-based surrogate model demonstrates strong agreement with the COMSOL results across all five output variables. The predicted values are closely aligned with the diagonal reference line, particularly for temperature, current density, and deformation, indicating high prediction accuracy on unseen data.

**Figure 12:**
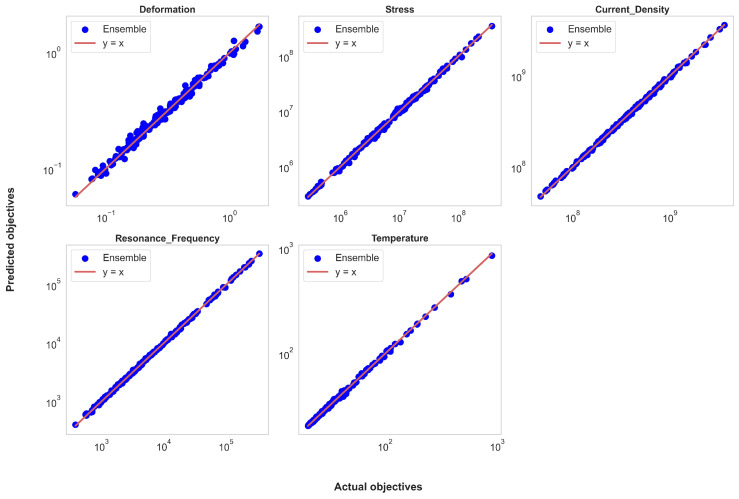
Parity plots: Ensemble-based surrogate model vs. COMSOL results (five output variables).

Figure 12 presents the corresponding parity plots for the Ensemble-based surrogate model. Although the Ensemble model captures the overall trends of the COMSOL results, its predictions show greater dispersion compared with GPR, especially near the low and high value ranges for some output variables. Larger deviations are observed for stress and temperature, suggesting lower local prediction accuracy in these regions. Nevertheless, considering its substantially lower training and inference time, the Ensemble model remains a practical alternative when computational efficiency is prioritized over maximum prediction accuracy.

#### 4.6.2. Absolute Relative Error Distribution Plot for Models

An Absolute Relative Error Distribution plot evaluates the predictive performance of the two surrogate models (GPR and Ensemble) across five output variables. These plots provide a comprehensive post-training assessment of surrogate model accuracy for the MEMS thermal actuator. The results are reported using the optimal preprocessing method selected for each output variable based on the MAPE criterion. To enable comparison among outputs with different physical units and scales, errors are reported as absolute relative errors.

**Figure 13:**
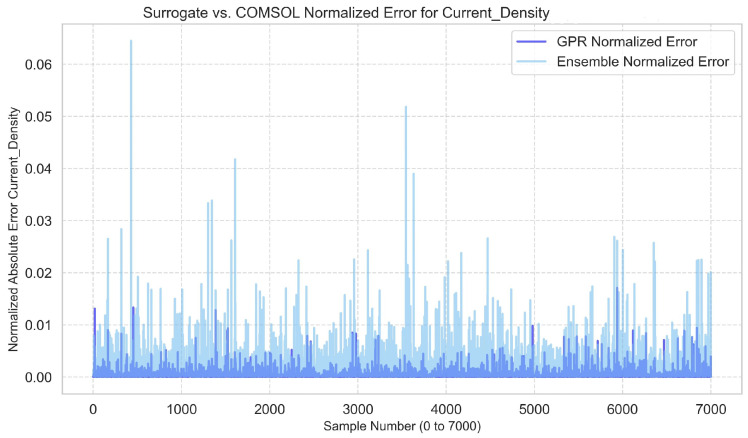
Absolute relative error distribution plots: Ensemble and GPR surrogate models vs. COMSOL results for Current Density.

**Figure 14:**
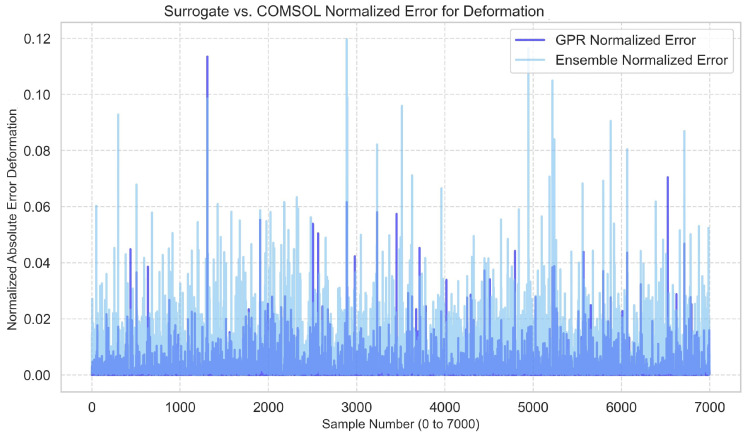
Absolute relative error distribution plots: Ensemble and GPR surrogate models vs. COMSOL results for Deformation.

**Figure 15:**
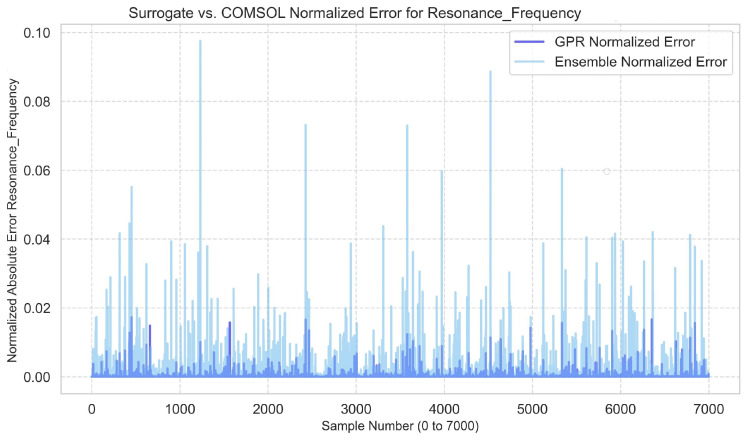
Absolute relative error distribution plots: Ensemble and GPR surrogate models vs. COMSOL results for Resonance_Frequency.

**Figure 16:**
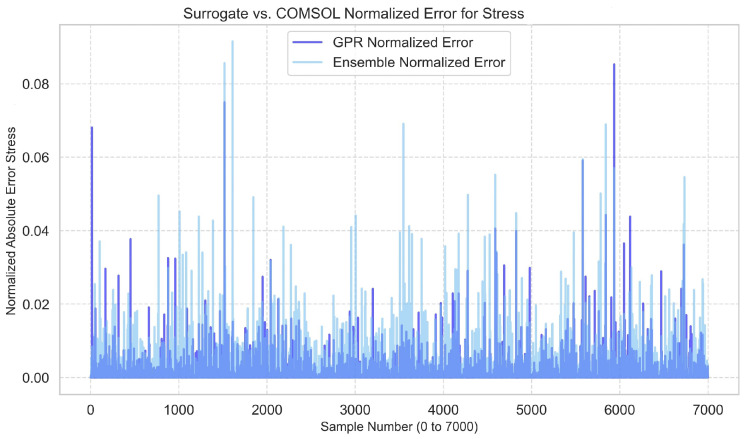
Absolute relative error distribution plots: Ensemble and GPR surrogate models vs. COMSOL results for Stress.

**Figure 17:**
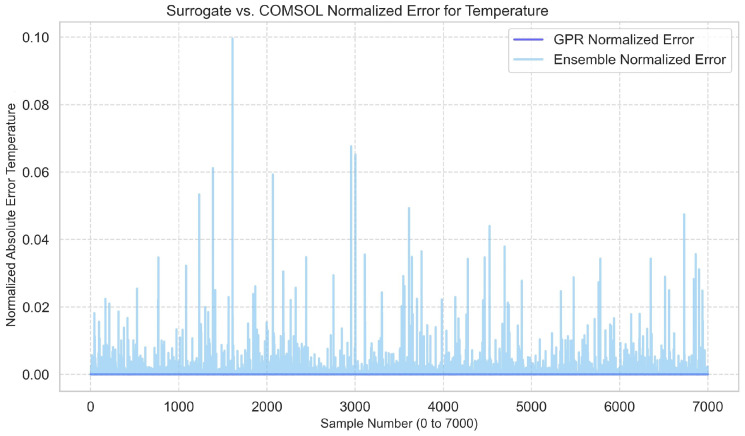
Absolute relative error distribution plots: Ensemble and GPR surrogate models vs. COMSOL results for Temperature.

- Current Density: Figure 13 compares the performance of the GPR and Ensemble models in predicting the current density of a MEMS thermal actuator. The GPR model demonstrates high accuracy and consistency, with errors mostly below 0.1% and a maximum error of 1.6%. In contrast, the Ensemble model exhibits greater fluctuations, with errors often below 0.2% but reaching up to 6% in some cases. Overall, GPR achieved lower prediction errors than the Ensemble model for current density.
- Deformation: Figure 14 illustrates the deformation prediction results, showing that the GPR model, despite a few local peaks (up to about 11%), achieved errors mostly below 2% and demonstrated more stable performance. In contrast, the Ensemble model exhibited greater variability, with errors primarily ranging from 2% to 6% and reaching up to around 12%. Overall, GPR provides higher accuracy and stability in predicting MEMS actuator deformation compared to the Ensemble model.
- Resonance Frequency: Figure 15 presents the resonance frequency predictions of the GPR and Ensemble models. The GPR model demonstrated high accuracy and stability, with over 90% of errors below 1% and a maximum observed error of about 3%. In contrast, the Ensemble model exhibited larger fluctuations, with errors reaching up to 10%. These results indicate that the GPR model achieves lower prediction errors for the resonant frequency of the MEMS thermal actuator.
- Stress: Figure 16 shows the normalized absolute error of stress predictions, comparing the performance of GPR and Ensemble models. The GPR model demonstrates high accuracy and stability, with the majority of errors below 1% and a maximum error of around 6.5%. In contrast, the Ensemble model exhibits larger and more fluctuating errors, with some peaks reaching approximately 8.5%. These results indicate that GPR provides better accuracy, stability, and generalization than Ensemble in predicting stress for the MEMS thermal actuator.
- Temperature: Figure 17 shows the normalized absolute error distribution for temperature predictions, comparing the performance of the GPR and Ensemble models for the MEMS thermal actuator. The GPR model exhibits a highly concentrated error distribution, with most prediction errors remaining below 1% and only a few isolated peaks reaching approximately 2.3%. In contrast, the Ensemble model demonstrates a wider error spread and larger fluctuations, where most errors remain below 2%, but several outliers rise up to nearly 7.5%. These results indicate that the GPR model provides more stable and accurate temperature predictions compared to the Ensemble approach, making it a more suitable surrogate model for MEMS thermal actuator modeling pipelines.

#### 4.6.3. Residual Error Distribution Plots for Ensemble and GPR Models

These plots show the distribution of residual errors (Actual vs. Predicted) for the Ensemble and GPR models. They facilitate a comparison of model accuracy, prediction bias, and error variability across the different output variables of the MEMS thermal actuator.

**Figure 18:**
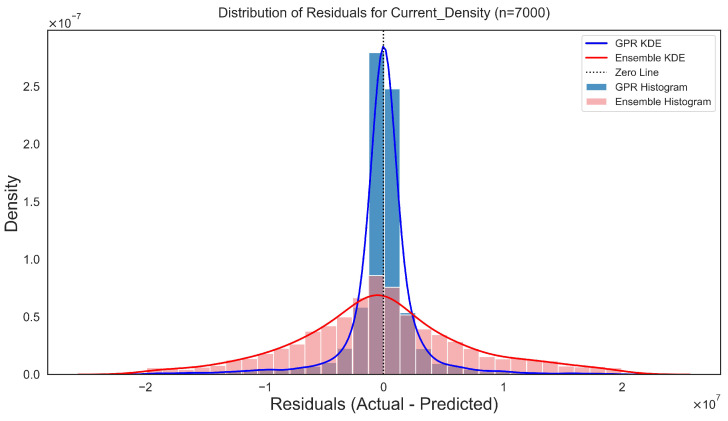
Residual error distribution of GPR and Ensemble surrogate models for Current Density.

**Figure 19:**
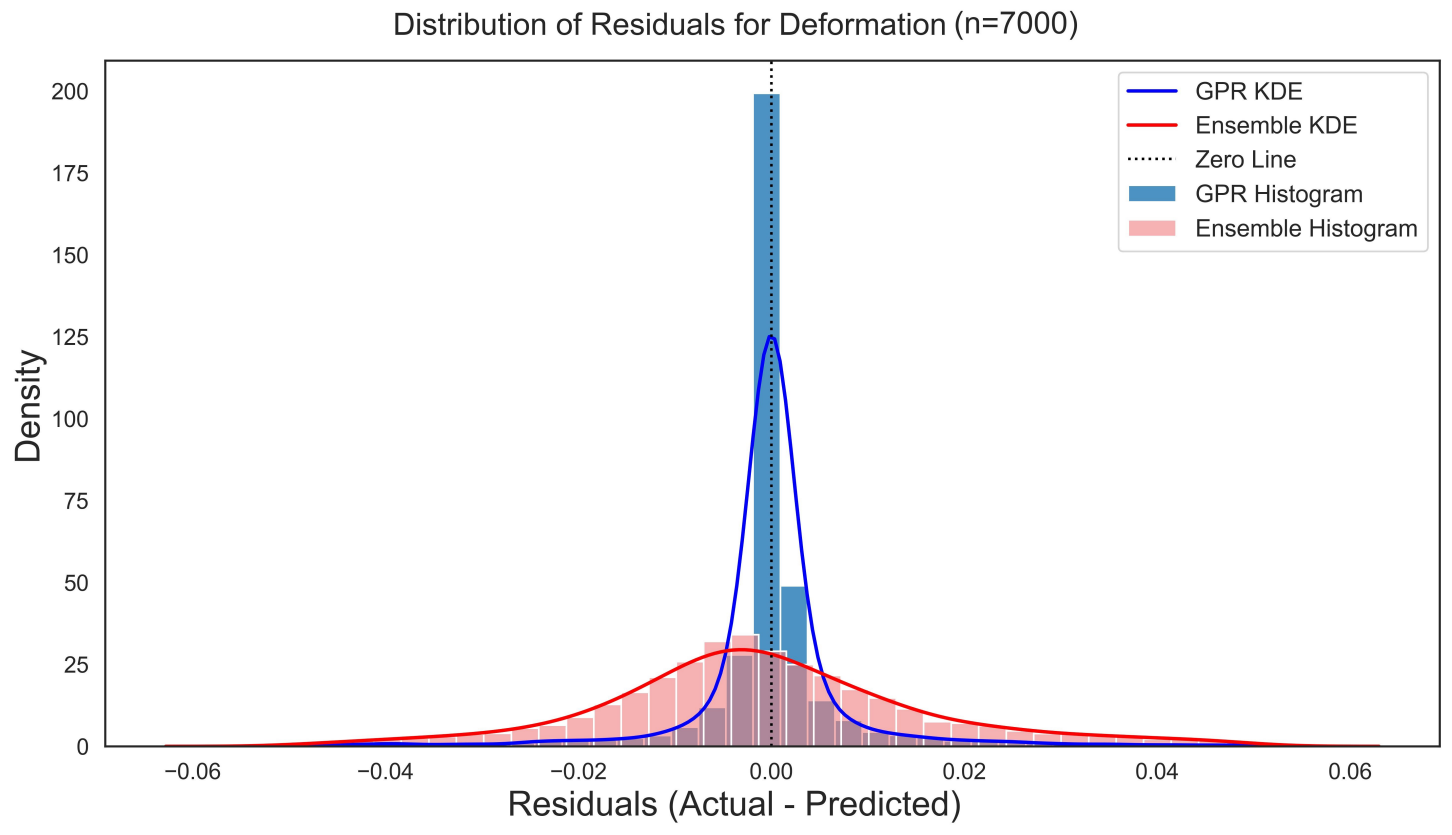
Residual error distribution of GPR and Ensemble surrogate models for Deformation.

**Figure 20:**
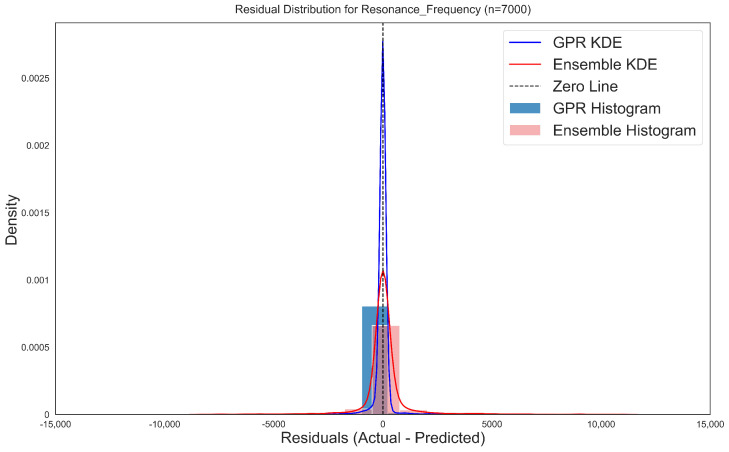
Residual error distribution of GPR and Ensemble surrogate models for Resonance_Frequency.

**Figure 21:**
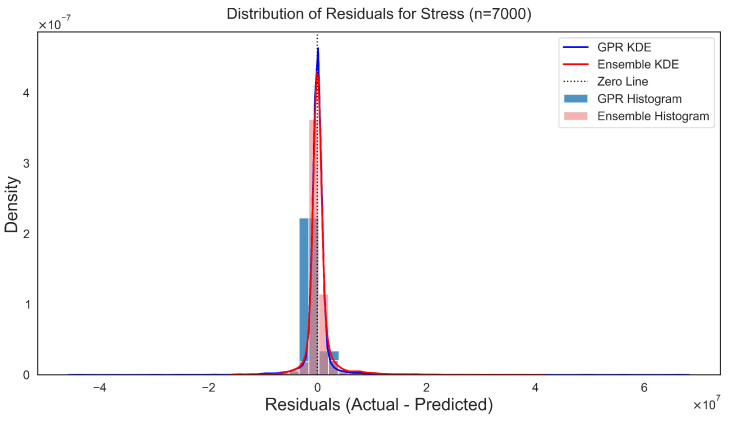
Residual error distribution of GPR and Ensemble surrogate models for Stress.

**Figure 22:**
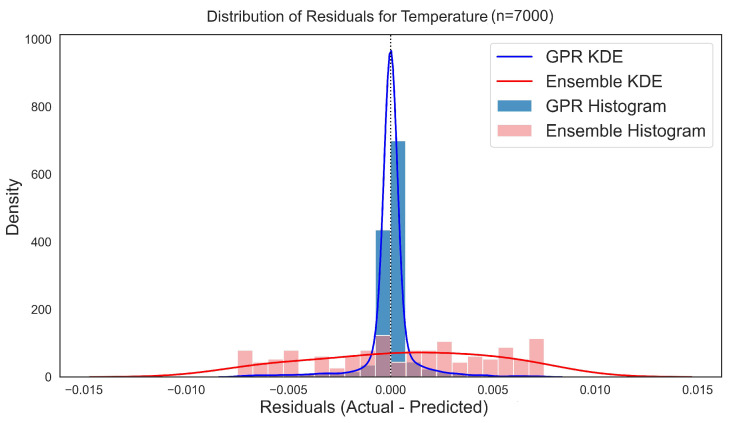
Residual error distribution of GPR and Ensemble surrogate models for Temperature.

- Current Density: Figure 18 presents the residual error distributions of the GPR and Ensemble models for current density prediction. The residuals of the GPR model are more closely clustered around zero than those of the Ensemble model, indicating lower prediction errors. These findings demonstrate the superior accuracy, stability, and agreement of the GPR model with the COMSOL data, suggesting that GPR is the more reliable method for current density prediction in MEMS actuator.
- Deformation: Figure 19 presents the residual error distributions of the GPR and Ensemble models for deformation prediction. The residuals of the GPR model are more closely clustered around zero than those of the Ensemble model, indicating superior predictive performance. Moreover, the narrower spread of the residuals demonstrates reduced error variability and enhanced reliability in deformation prediction.
- Resonance Frequency: Figure 20 shows the residual error distributions of the GPR and Ensemble models for resonance frequency prediction. The residuals of the GPR model are more tightly concentrated around zero, indicating lower prediction errors than those of the Ensemble model.
- Stress: Figure 21 compares the residual distributions of the GPR and Ensemble models for stress prediction. Both models are centered around zero, indicating the absence of significant prediction bias. However, the GPR model exhibits a sharper and narrower peak at zero, demonstrating that a larger proportion of its prediction errors are concentrated near the true values. In contrast, the Ensemble model shows a broader distribution with heavier tails, indicating greater variability in its prediction errors. These findings highlight the superior accuracy and consistency of the GPR model for stress prediction, while the Ensemble model may still be advantageous for highly heterogeneous datasets.
- Temperature: Figure 22 shows that the GPR model exhibits lower residual errors in temperature prediction. The residual distribution for GPR is narrower and more concentrated around zero compared to the Ensemble model, indicating higher precision and stability. These results confirm the robustness of GPR in temperature modeling. Nevertheless, the Ensemble model may still be advantageous for datasets with higher variability or complex patterns.

### 4.7. Evaluation of the Proposed Method

Previous studies on alternative modeling for MEMS actuators have primarily focused on GPR methods, which have a limited prediction scope, typically addressing only one or two target variables and using simple, uniform normalization for small FEM datasets [4,9,10,11]. These limitations reduce scalability, diminish the robustness of outputs, and restrict applicability in high-dimensional, multi-objective design spaces. To overcome these challenges, the proposed framework offers an enhanced approach that can more effectively handle multiple target variables, demonstrating superior performance compared to previous models.

In the present study, the scaling was investigated on a much larger dataset, including five different output variables. Unlike the previous surrogate-assisted optimization studies by Nazari et al. [10,11], where the authors applied a single scaling method to all targets, the proposed method selected the best scaling specifically for each objective. It is important to note that in the present work, the normalization was performed in the range of 0 to 1. In this study, the results showed that the Log + MinMax normalization method performed best only for the stress and deformation objectives, while the other three output variables performed better with different scaling methods. This indicates that using a dedicated scaling for each objective, especially in situations with large data volumes and diverse values, has a significant advantage over using a single method for all output variables.

Furthermore, the present study introduces a versatile surrogate modeling pipeline for thermal MEMS actuators that uses GPR and an ensemble model to predict five output variables (deformation, stress, temperature, current density, and resonance frequency). With 10,000 samples and eight advanced preprocessing techniques, the GPR model achieved MAPEs ranging from 0.81% to 2.58% across all output variables, including 0.81% for temperature and 2.58% for deformation, thereby improving accuracy and stability. The ensemble model also achieved MAPEs ranging from 3.05% to 9.20% across all output variables, including 3.05% for current density and 9.20% for stress. Additionally, we employed 5-fold cross-validation to evaluate the models’ performance. Although this approach increased the accuracy of the evaluation and reduced the likelihood of overfitting, it resulted in longer training and evaluation times compared to previous methods. The ensemble model training time of 5.6 min for 10,000 samples also improved the computational efficiency compared to previous smaller-sample approaches. The findings emphasize the importance of choosing appropriate scaling methods and combining them to improve the performance of surrogate models. In addition to prediction accuracy, the Gaussian Process Regression model provides predictive uncertainty in the form of standard deviation. This allows for the quantification of confidence in each prediction, which is particularly valuable in MEMS design for identifying regions of higher reliability and supporting conservative design decisions, and identifying cases where additional FEM verification may be needed. In the present study, increasing the number of data points to 10,000 samples will also be very useful in future developments of the actuator’s design. With this volume of data points, the necessary calculations for this scale have already been performed, and the model can be extended to accommodate future changes in actuator design, thus achieving flexibility and scalability in future research.

## 5. Conclusions

In this study, a comprehensive surrogate model was developed to simulate the performance of MEMS thermal actuators using Gaussian Process Regression (GPR) and a hybrid model (Ensemble) that is capable of simultaneously predicting five key variables (deformation, stress, temperature, current density, and resonance frequency) with high accuracy. Using 10,000 samples generated by Latin Hypercube Sampling and COMSOL simulation and applying eight preprocessing methods, the proposed models showed that advanced combinations such as Log + MinMax, Log + Robust, and Yeo–Johnson improve accuracy: GPR achieved MAPE ≈0.81–2.58%, whereas the Ensemble achieved MAPE ≈3.05–9.20% depending on the objective.

To ensure reliability and reduce the risk of overfitting, 5-fold cross-validation was applied during training. While this increased overall computational time, it improved the robustness of the results. An analysis of computational costs for 10,000 samples revealed that the majority of time was dedicated to FEM simulation. Specifically, FEM simulation accounted for 27.75 h, GPR training for 1861.34 min, and Ensemble training for 5.61 min. Thus, the combined computational time for FEM and GPR training totaled 58.77 h, making the additional Ensemble training time (27.84 h) comparatively negligible.

Despite its promising results, the current study has some limitations. Specifically, the model assumes constant material properties and does not account for anchor or boundary effects, factors that could influence stress distribution and deformation in practical MEMS devices. Future research should incorporate temperature-dependent material properties, account for anchor and boundary effects, and apply multi-objective optimization algorithms (e.g., NSGA-II or Bayesian optimization) to further enhance actuator performance. Additionally, advanced surrogate modeling approaches, such as hybrid CNN-GPR frameworks or multi-output deep neural networks (DNNs), could be explored to predict all output variables simultaneously, although these methods may increase computational complexity.

Generating 10,000 FEM samples and training the GPR model demanded substantial computational effort, underscoring the need for efficient surrogate modeling strategies. Nevertheless, the proposed framework effectively accelerates the design and analysis of MEMS thermal actuators at early development stages, providing a foundation for more advanced, multi-objective actuator designs with improved predictive accuracy.

## Figures and Tables

**Table 1:** Literature Summary Table.

Prior Work Category and Representative Studies	Typical Setup	Main Difference Relative to This Work	How the Present Work Differs
CNN-based surrogate models [9]	CNN prediction of torque/radial force for electrostatic MEMS motors	Strong performance, but it depends on structured spatial inputs and is less transferable to general tabular design	Uses tabular design variables for a thermal MEMS actuator and predicts five outputs
GPR-based predictive models for MEMS devices [4]	GPR models for micro-actuators/resonators with limited variables or outputs	Good predictive capability but limited variable and objective scope	Extends to a broader nonlinear space with simultaneous prediction of five output variables
Surrogate-assisted optimization frameworks [10]	Surrogate model integrated with NSGA-II or similar evolutionary search	Surrogate assessed mainly within the optimisation loop; constrained design-space setting	Positions the surrogate as a standalone predictive tool as well as a basis for later optimisation
Adaptive-sampling frameworks [11]	Limited-sample adaptive sampling with uniform preprocessing	Limited data range and uniform preprocessing may reduce robustness for heterogeneous outputs	Uses 10,000 samples and output-specific preprocessing strategies
This study	10,000 COMSOL samples; GPR and Ensemble models; five outputs	—	Multi-output, scalable, standalone predictive framework with output-specific preprocessing

**Table 2:** Ranges of Design Variables.

Design Variable	Symbol	Lower Bound	Upper Bound	Unit
Length of the hot arm	$L_{1}$	500	10,000	μm
Length of the thick part of the cold arm	$L_{2}$	100	7000	μm
Length of the thin part of the cold arm (flexure)	$L_{3}$	150	8000	μm
Length of the junction	$L_{j}$	10	100	μm
Thickness of the device	$t_{\mathrm{all}}$	5	5	μm
Width of the hot arm	$W_{1}$	10	200	μm
Width of the thick part of the cold arm	$W_{2}$	20	1000	μm
Width of the thin part of the cold arm	$W_{3}$	10	200	μm
Width of the junction	$W_{j}$	10	100	μm
Applied Voltage to the actuator	AV	5	5	V

**Table 3:** Output Variables and Optimization Goals.

Output Variables	Unit	Goal
Deformation	μm	Maximize
Stress	Pa	Minimize
Temperature	°C	Minimize
Current Density	A/m^2^	Minimize
Resonance Frequency	Hz	Maximize

**Table 4:** Comparison of sampling methods for surrogate modeling.

Method	Advantages	Disadvantages
Simple Random Sampling	Easy to implement; unbiased sampling	Poor space coverage; clustering may occur
Monte Carlo Sampling	Statistically sound; simple	Requires large sample size for uniform coverage
Full Factorial Design	Complete coverage of factor combinations	Exponential growth with dimensionality (curse of dimensionality)
Latin Hypercube Sampling (LHS)	Efficient space-filling; ensures stratification; good performance with limited samples	No guaranteed optimal spacing unless optimized (e.g., maximin criterion)
Sobol Sequence	Low-discrepancy; better uniformity than random sampling	More complex implementation; may not preserve independence

**Table 5:** Comparison of Ensemble strategies based on test-set MAPE (%) and training time.

Output Variables	Simple Avg.	Weighted Avg.	Boosting	Stacking
Current Density	3.05	4.03	3.80	3.51
Stress	9.20	14.68	12.83	19.31
Deformation	6.85	9.99	9.14	8.26
Temperature	3.79	3.89	3.95	4.05
Resonance Frequency	4.39	6.38	5.89	7.82
Average MAPE	5.46	7.79	7.12	8.59
Training Time (min)	5.61	7.12	7.35	8.58

**Table 6:** GPR vs. Ensemble Models: Performance and Relative Improvement Across Varying Sample Sizes.

Output Variables	GPR (5 k Samples)	GPR (10 k Samples)	Improvement (GPR)	Ensemble (5 k Samples)	Ensemble (10 k Samples)	Improvement (Ensemble)
Current Density	1.30%	1.03%	0.27%	3.91%	3.05%	0.86%
Deformation	4.11%	2.58%	1.53%	8.76%	6.85%	1.91%
Temperature	1.20%	0.81%	0.39%	4.63%	3.79%	0.84%
Resonance Frequency	2.25%	1.53%	0.72%	5.41%	4.39%	1.02%
Stress	4.70%	1.53%	3.17%	10.61%	9.20%	1.41%
Average	2.71%	1.50%	1.21%	6.66%	5.46%	1.20%

**Table 7:** Complete computational cost breakdown: FEM simulation (hours), model training (minutes), and total time (hours).

Samples	FEM Time (h)	Ensemble Training (min)	GPR Training (min)	Total (FEM + Ens.) (h)	Total (FEM + GPR) (h)
100	0.28	0.63	2.03	0.29	0.32
500	1.38	0.78	9.53	1.39	1.54
1000	2.75	1.12	20.86	2.76	3.09
2000	5.53	1.75	38.39	5.56	6.17
3000	8.23	2.21	83.52	8.26	9.62
4000	10.92	2.71	189.62	10.97	14.08
5000	13.68	3.23	270.49	13.74	18.19
6000	16.48	3.79	374.37	16.54	22.72
7000	19.28	4.13	849.63	19.35	33.44
8000	22.11	4.78	1260.42	22.19	43.12
9000	24.93	5.19	1289.75	25.02	46.43
10,000	27.75	5.61	1861.34	27.84	58.77

**Table 8:** Inference time and MAPE comparison of Ensemble and GPR models (2000 samples).

Model	Time (s)	Deformation MAPE	Stress MAPE	Temperature MAPE	Current Density MAPE	Resonance Frequency MAPE
Ensemble	0.206	11.11	9.67	3.73	5.08	6.13
GPR	16.315	2.50	5.09	0.74	1.22	2.31

## Data Availability

The original contributions presented in this study are included in the article. Additional data supporting the findings of this study are available from the corresponding author upon reasonable request.
